# Unveiling the Influence of Hot Carriers on Photovoltage Formation in Perovskite Solar Cells

**DOI:** 10.3390/ma18010085

**Published:** 2024-12-28

**Authors:** Muhammad Mujahid, Aurimas Čerškus, Jonas Gradauskas, Asta Grigucevičienė, Raimondas Giraitis, Konstantinas Leinartas, Andžej Lučun, Kazimieras Petrauskas, Algirdas Selskis, Algirdas Sužiedėlis, Aldis Šilėnas, Edmundas Širmulis, Steponas Ašmontas

**Affiliations:** 1 Laboratory of Electronic Processes, Center for Physical Sciences and Technology, Saulėtekio Ave. 3, LT-10257 Vilnius, Lithuania; muhammad.mujahid@ftmc.lt (M.M.); aurimas.cerskus@ftmc.lt (A.Č.); jonas.gradauskas@ftmc.lt (J.G.); andzej.lucun@ftmc.lt (A.L.); kazimieras.petrauskas@ftmc.lt (K.P.); algirdas.suziedelis@ftmc.lt (A.S.); aldis.silenas@ftmc.lt (A.Š.); edmundas.sirmulis@ftmc.lt (E.Š.); 2 Department of Electrochemical Material Science, Center for Physical Sciences and Technology, Saulėtekio Ave. 3, LT-10257 Vilnius, Lithuania; asta.griguceviciene@ftmc.lt (A.G.); konstantinas.leinartas@ftmc.lt (K.L.); 3 Department of Characterization of Materials Structure, Center for Physical Sciences and Technology, Saulėtekio Ave. 3, LT-10257 Vilnius, Lithuania; raimondas.giraitis@ftmc.lt (R.G.); algirdas.selskis@ftmc.lt (A.S.)

**Keywords:** advanced photoelectric materials, perovskite solar cell, transient photovoltage, thin film, hot carriers

## Abstract

The experimental and theoretical study of photovoltage formation in perovskite solar cells under pulsed laser excitation at 0.53 μm wavelength is presented. Two types of solar cells were fabricated on the base of cesium-containing triple cation perovskite films: (1) Cs_x_(FA_0.83_MA_0.17_)_(1−x)_Pb(I_0.83_Br_0.17_)_3_ and (2) Cs_x_(FA_0.83_MA_0.17_)_(1−x)_Pb_0.8_Sn_0.2_(I_0.83_Br_0.17_)_3_. It is found that photovoltage across the solar cells consists of two components, *U* = *U_ph_* + *U_f_*. The first one, *U_ph_*, is the traditional photovoltage arising due to laser radiation-induced electron-hole pair generation. The second one, *U_f_*, is the fast component following the laser pulse and has a polarity opposite to that of *U_ph_*. It is shown that the fast photovoltage component results from the laser radiation-caused heating of free carriers. The transient photovoltage measurements show that the values of the fast component *U_f_* are nearly the same in both types of perovskite solar cells. The magnitude of the traditional photovoltage of mixed Pb-Sn perovskite solar cells is lower than that of Pb-based cells.

## 1. Introduction

The organic-inorganic hybrid perovskite based solar cells (PSCs) are gaining attention as strong candidates for the future of solar technology, which is attributed to their wide spectral response and impressive photocurrent output [1,2]. Over the past 10 years, the power conversion efficiency (PCE) of PSCs has significantly increased from 3.8% to over 25.7% [3,4], rendering them highly appealing as renewable energy sources that contribute to reduced CO_2_ emissions. The impressive efficiency of PSCs can be linked to the distinctive interplay of optical and electrical characteristics found in perovskite films. This includes robust light absorption throughout the full visible spectrum [5], notable defect tolerance [6], higher carrier mobility [7,8,9], extended carrier diffusion length [10,11], and prolonged lifetimes of the generated charge carriers [12,13,14]. To enhance the quality of perovskite films, various techniques are used, including hot casting [15], additive engineering [16], rapid thermal annealing [17], antisolvent engineering [18], sequential deposition [19], vacuum thermal evaporation [20], and additive-assisted deposition [21,22] have been developed. Enhancements in charge carrier management, intricately linked to the fill factor and open-circuit voltage, present a viable avenue for boosting the performance of PSCs [23]. Despite much effort, the performance of the best PSCs until now is lower than the predicted theoretical Shockley–Queisser limit [24,25,26]. Several reasons reduce the PCE: non-radiative recombination losses [25,27,28,29,30], optical losses due to light reflection [31,32], thermal effects [33]. The conversion efficiency of a solar cell is influenced by the effective utilization of photons with energies near the forbidden-energy gap [34]. The photons with elevated energies generate electron-hole pairs, and the surplus energy is imparted to the free carriers, effectively transforming them into hot carriers. The electromotive force generated by hot carriers in a single junction solar cell is contrary to the polarity of the photovoltage produced from the generation of electron-hole pairs [35]. Therefore, the efficiency of a single junction solar cell is reduced by the light-induced carrier heating. In perovskite solar cells, the electromotive force of hot carriers can also be generated because there are energy band bendings and corresponding potential barriers near the charge transport layers. When heated, the carriers may gain sufficient energy to overcome the barriers, and the hot carrier thermoelectromotive force will be generated across a PSC. The value of hot carrier thermoelectromotive force, as in the case of the Schottky junction, is directly proportional to the height of the potential barrier of the band bending near the charge transport layers and the heating of the carriers [36,37]. In addition, slow hot carrier cooling in perovskites [38,39,40,41] promotes the formation of the thermoelectromotive force of hot carriers. Nevertheless, the impact of hot carriers on the generation of photovoltage in PSCs remains unexplored to date.

The new concept of a hot carrier solar cell was proposed by Ross and Nozik [42]. In this context, it is essential to extract hot carriers through the limited energy-selective contacts at a pace that exceeds the rate at which they transfer their energy to the lattice. Theoretically, hot carrier solar cells have the potential to exceed the Shockley–Queisser limit, achieving efficiencies of up to 66%. A significant number of theoretical and experimental studies have been conducted for the advancement of hot carrier solar cells [43,44,45,46,47,48,49,50,51,52,53,54]. Nonetheless, a functional hot carrier solar cell suitable for practical applications has yet to be developed.

This paper provides an experimental investigation into the structural and optical characteristics of two varieties of perovskite films Cs_0.1_(FA_0.83_MA_0.17_)_0.9_Pb(I_0.83_Br_0.17_)_3_ and Cs_0.1_(FA_0.83_MA_0.17_)_0.9_Pb_0.8_Sn_0.2_(I_0.83_Br_0.17_)_3_, and investigates photoelectric properties of solar cells fabricated on their base. The transient photovoltage measurements made it possible to detect the electromotive force of hot carriers, which essentially changes the kinetics of photovoltage formation in the PSCs exposed to laser radiation.

## 2. Fabrication and Characterization of Perovskite Films

**Figure 1:**
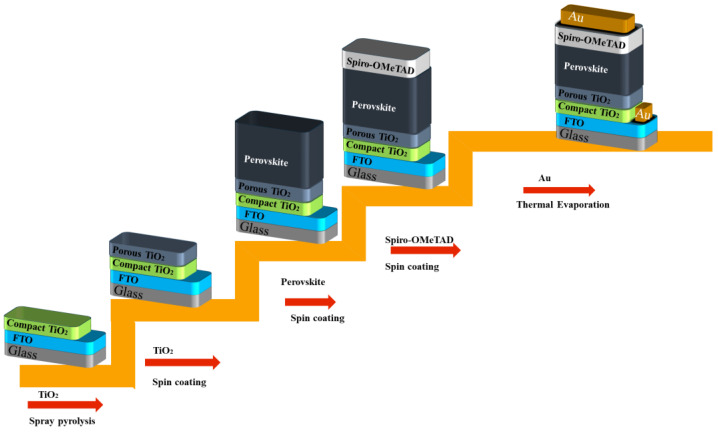
Schematic representation of the development process of perovskite solar cells illustrated in a progressive manner.

In this study, perovskite films were produced in accordance with the previously outlined methods as detailed in the referenced papers [55,56,57]. The articles delivered provided an in-depth examination of the reagents, detailing their concentrations and purity levels, alongside the sequence of procedures and equipment employed in the fabrication process of the Cs_0.1_(FA_0_._83_MA_0.17_)_0.9_Pb(I_0_._83_Br_0.17_)_3_ PSC [58]. The deposition process for triple cation perovskite films was performed on glass substrates with dimensions of 25 × 25 mm^2^, which were coated with a transparent layer of fluorine-doped tin oxide (FTO) (TEC 10, Ossila B.V., Sheffield, UK). Following this, around 7 × 25 mm^2^ of the FTO coating was eliminated from one edge of the glass substrate using zinc powder (Sigma-Aldrich, St. Louis, MO, USA) and hydrochloric acid (Merck KGaA, Darmstadt, Germany). A 20 min sonication was subsequently conducted in a 2% Hellmanex solution (Sigma-Aldrich, USA). The substrates underwent rinsing with deionized water followed by sonication in isopropanol (Merck KGaA, Darmstadt, Germany) for a duration of 20 min, preceding a plasma treatment lasting 10 min. Figure 1 presents a schematic illustration of the principal stages of fabricating perovskite solar cells. In the initial step, a compact layer of TiO_2_ (Greatcell-Solar-Italia S.R.L., Rome, Italy) with an approximate thickness of 30 nanometers was applied onto the FTO coating via spray pyrolysis of a precursor solution containing titanium diisopropoxide (bis) acetylacetonate Ti(acac)_2_OiPr_2_ (Merck-KGaA, Darmstadt, Germany) and anhydrous ethanol (Sigma-Aldrich, MO, USA) in a ratio of 1:9 by volume. The process was carried out at 450 °C and then subjected to 15 min of annealing. After the deposition process, the structures had enough time to reach room temperature. In step II, a mesoporous TiO_2_ layer was formed through the spin coating of 180 μL of a suspension containing titanium oxide nanoparticles (30 nm) diluted in ethanol at a 1:6 weight ratio. This was applied at 4000 rpm for 20 s, with an acceleration of 2000 rpm∙s^−2^. The substrates underwent annealing at 450 °C for a duration of 30 min. After the cooling process, the samples were transferred to a glove box that was filled with nitrogen (M-Braun Inertgas-Systeme GMBH, Garching bei München, Germany). In step III, the perovskite layer was deposited from a freshly prepared solution that included 1.2 M lead iodide (Sigma-Aldrich, MO, USA), 0.2 M methylammonium bromide (Greatcell Solar Italia S.R.L., Italy), 0.2 M lead bromide (Sigma-Aldrich, MO, USA), and 1 M formamidinium iodide (Greatcell-Solar Italia S.R.L., Italy). A solvent was formulated by combining anhydrous N,N-dimethylformamide (Sigma-Aldrich, MO, USA) with dimethylsulfoxide (DMF/DMSO) (Sigma-Aldrich, MO, USA) in a volume ratio of 4:1. The concentration of cesium ions in the precursor solution was modified to 10% through the addition of the suitable quantity of CsI (Strem-Chemicals INC MS, Newburyport, MA, USA) (1.5 M of CsI in DMSO). When producing the Cs_0.1_(FA_0_._83_MA_0.17_)_0.9_Pb_0_._8_Sn_0_._2_(I_0_._83_Br_0.17_)_3_ perovskite film, the solution of PbI_2_ and SnI_2_ in molar ratio 4:1 was added to the precursor. Until all components were fully dissolved, the precursor solutions were mixed at 60 °C for one hour, resulting in the formation of Cs_0.1_(FA_0.83_MA_0.17_)_0.9_Pb_0.8_Sn_0.2_(I_0.83_Br_0.17_)_3_ perovskite. A two-step program was implemented to spin-coat the prepared precursor solution: the initial stage involved a speed of 1000 rpm for 10 s, followed by a second stage at 6000 rpm for 30 s. The upper surface underwent treatment with 150 μL of chlorobenzene (Sigma Aldrich, MO, USA) for approximately 10 s prior to the cessation of spinning, followed by annealing at 100 °C for a duration of 60 min.

Formation of the hole-transporting layer (HTL) was the fourth step in the cell fabrication. The HTL was formed by spinning 150 μL of 70 mM solution of 2-N,-2-N,2-N′-,2-N′,7-N,7-N,7-N′,7-N′-octakis(4-methoxyphenyl)-9,-9′-91-spiro-bi[fluorene]-2,-2′,-7,-7′-tetramine (Spiro-OMeTAD) in chloro-benzene (85.78 mg/mL), supplemented with 17 μL of Li-bis((trifluoromethyl)sulfonyl)im-ide (Li-TFSI) salt in anhydrous-acetonitrile (520 mg/mL) and 28.8 μL of 4-tert-butylpyri-dine (TBP) solutions just before the application (all from Sigma Aldrich, MO, USA). The dopants for Li-TFSI and TBP were added to the Spiro-OMeTAD at a molar ratio of 0.5 and 3.3, respectively. The spin-coating phase of step IV was sustained at 4000 rpm for 25 s with an acceleration of 2000 rpm∙s^−2^. In the last step, V, about 70 nm-thick gold contacts were deposited on Spiro-OMeTAD and FTO using thermal evaporation in the vacuum chamber of the “VAKSIS-PVD-Vapor-5S_Th” (Vaksis-Research and Development & Engineering, Ankara, Turkey).

Using a scanning electron microscope (SEM) (Helios NanoLab 650, FEI, Hillsboro, OR, USA), the thickness and morphology of the perovskite films were examined. Combining the SEM with an energy dispersive X-ray spectrometer (EDX) (INCA Energy, Oxford Instruments, Abingdon, UK), we were able to determine the films’ chemical composition. A theta/theta goniometer and an X-ray diffractometer (SmartLab, Rigaku, Tokyo, Japan) were used to analyze the crystalline structure of the perovskite films that were manufactured. The XRD has a 9 kW power rotating Cu anode X-ray source. The Bragg-Brentano geometry was used to measure the patterns within a 2θ range of 10–70°. As in our previous work [59], we analyzed the primary characteristics of the XRD patterns of full-scale perovskite layers and also conducted a detailed analysis of the XRD pattern fragments within the 2θ range of 26–29°.

Photoluminescence (PL) and transient PL spectra were measured at ambient temperature. The radiation of a pulsed laser was used for the excitation with wavelength 532 nm at 10 µJ cm^−2^ (Standa Ltd., Vilnius, Lithuania). The data were recorded using a 1 m monochromator and a thermoelectrically cooled photomultiplier tube (PMT) that used a photon counting technique. Our earlier study provided more details on the technique [58].

Investigating the transient photovoltage of the solar cells was undertaken using a diode-pumped frequency-doubled Nd: YAG-LBO-laser-NL202 (Ekspla Ltd., Vilnius, Lithuania). The laser pulses were 9 ns long and had a wavelength of 532 nm, generated at a repetition rate of 50 Hz. Optical power meter PM400 by Thorlabs Inc. of Newton, NJ, USA, was used to measure the average power of the laser radiation. For the transient photovoltage and laser pulse recordings, the team consulted Agilent Technologies’ digital storage oscilloscope (Santa-Clara, CA, USA) and Standa’s high-speed optical signal reference detector (Vilnius, Lithuania), respectively.

The absorbance spectra of the perovskite films were estima ted using the optical transmission data acquired with the spectrometer AvaSpec-ULS2048XL-EVO (Avantes, Apeldoorn, Netherlands). The current–voltage characteristics of the solar cells were measured directly using 2602A equipment (Keithley Instruments Inc., Cleveland, OH, USA). A Newport model 67005 spectrum lamp (Newport Corp., Irvine, CA, USA), fixed at the right distance, produced 100 mW/cm^2^ of irradiance.

## 3. Results and Discussion

**Figure 2:**
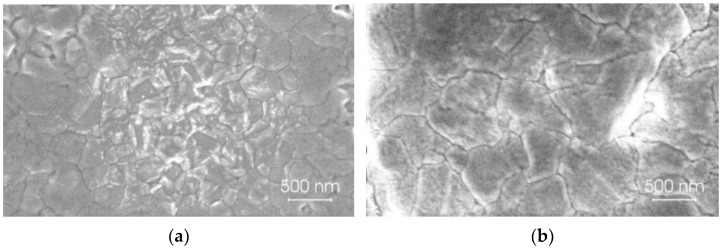
Top-view SEM images of the formed perovskite films: (**a**) Pb-based perovskite; (**b**) Pb-Sn based perovskite.

The top-view SEM images of two type perovskite films Cs_0.1_(FA_0_._83_MA_0.17_)_0.9_Pb(I_0_._83_Br_0.17_)_3_ and Cs_0.1_(FA_0_._83_MA_0.17_)_0.9_Pb_0_._8_Sn_0_._2_(I_0_._83_Br_0.17_)_3_ are shown in Figure 2. The perovskite films have comparable grain sizes and morphologies. Both films have smooth, pinhole-free surfaces, as has been noted before [60,61,62]. The grain sizes of the Pb-based perovskite films are smaller than those of the mixed metal Pb-Sn halide perovskite. As is known [62], the typical grain size is proportional to the perovskite layer thickness.

**Figure 3:**
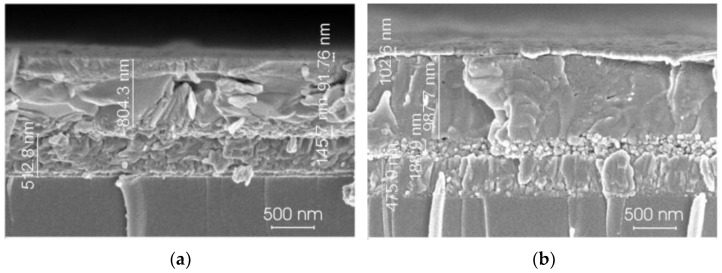
Cross-sectional SEM images of the formed perovskite films: (**a**) Pb-based perovskite; (**b**) Pb-Sn based perovskite.

Figure 3 illustrates the cross-sectional SEM images of the identical films. The thickness of the Pb-Sn based perovskite film surpasses that of the Pb-based perovskite film. In the lead-based perovskite, the grains exhibit a tendency to stack upon one another, whereas in the Pb-Sn perovskite, the grains are larger and predominantly organized in a lateral arrangement.

**Figure 4:**
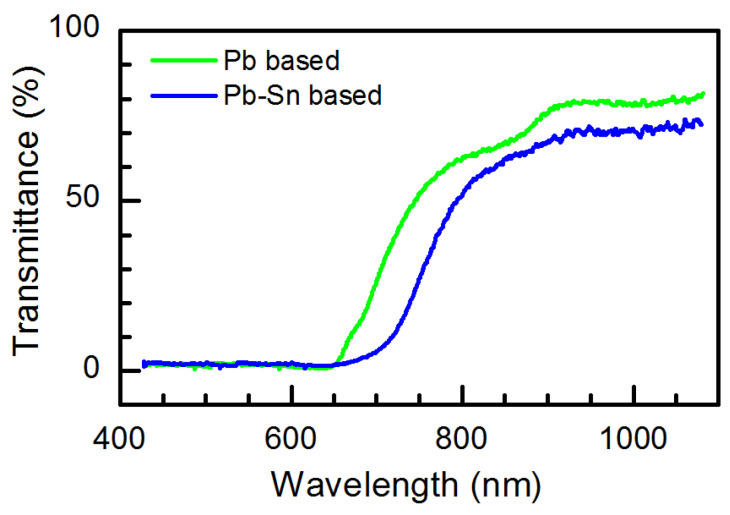
Optical transmittance spectra of Pb-based and Pb-Sn based perovskite films.

The transmittance spectra of the Pb-based perovskite and Pb-Sn based perovskite films are presented in Figure 4. It is seen that the transparency of the Pb-Sn based perovskite in the infrared range is lower than that of pure lead-based perovskite. Introducing tin into the perovskite layer reduces the forbidden energy gap [63], and therefore, a redshift of the transmittance is observed.

**Figure 5:**
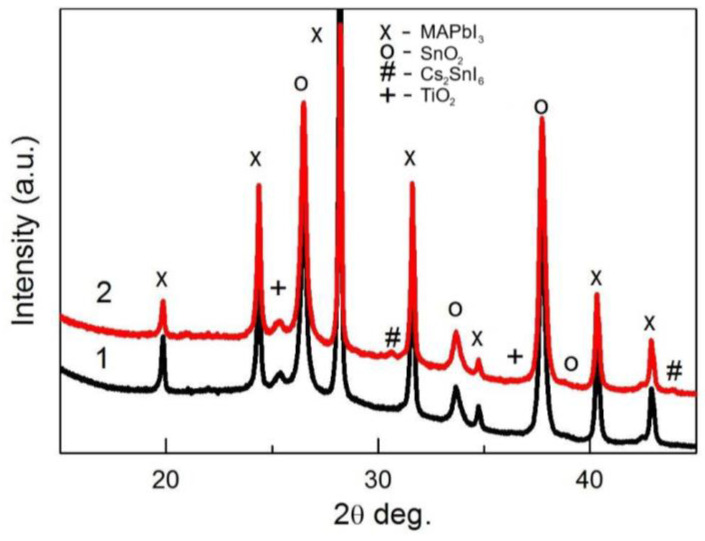
The XRD patterns of perovskite films on TiO_2_/FTO/glass substrate: pattern 1 (black)—without Sn; pattern 2 (red)—with the addition of 20% SnI_2_. Patterns were measured using the Bragg-Brentano method.

The XRD patterns of perovskite films fabricated using the solution without SnI_2_ (pattern 1, black) and with 20% of SnI_2_ and 80% of PbI_2_ (pattern 2, red) are presented in Figure 5. The patterns measured using the Bragg-Brentano method provide information not only from the perovskite film but also from the FTO (peaks labelled by “o”). Small peaks, visible on pattern 2 and labelled with the sign “#”, can be attributed to the Cs_2_SnI_6_ phase. A small peak at a 2theta angle of about 25.5 degrees should correspond to TiO_2_ (anatase) deposited on FTO before forming the perovskite.

**Figure 6:**
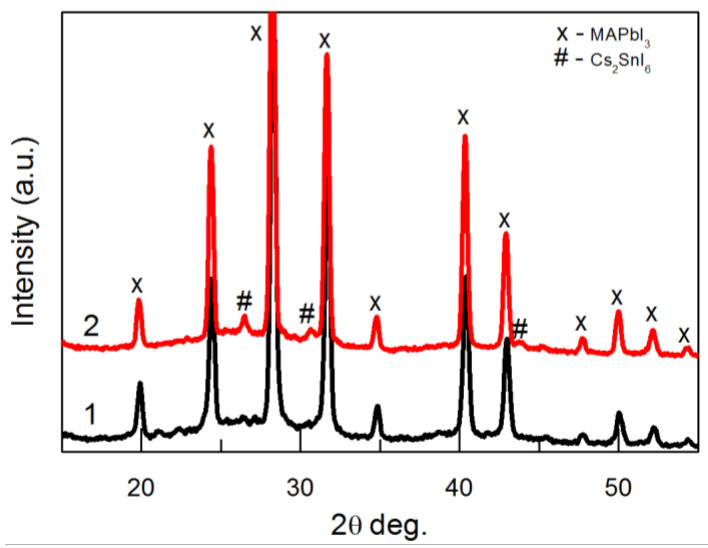
The XRD patterns of perovskite films on TiO_2_/FTO/glass substrate: pattern 1 (black)—without Sn; pattern 2 (red)—with the addition of 20% SnI_2_. Patterns were measured using the grazing incidence method.

Figure 6 shows the XRD patterns of the same perovskite samples measured using the grazing incidence (GIXRD) method (the angle between the incident X-ray beam and the sample surface is 0.5 degrees). This method yields information from the topmost volume (200–300 nm) of the perovskite. Only perovskite peaks are visible on pattern 1, i.e., in the sample fabricated without SnI_2_. Pattern 2 presents three peaks corresponding to the Cs_2_SnI_6_ phase. This suggests that this phase is formed mainly in the upper layers of the perovskite.

**Figure 7:**
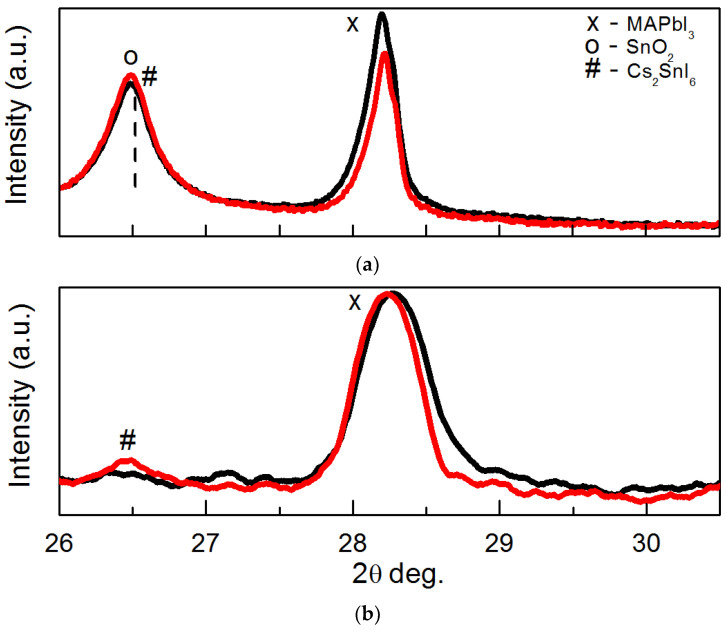
Fragments of XRD patterns measured in Bragg-Brentano (**a**) and GIXRD (**b**) methods. Patterns for perovskite formed without SnI_2_ are depicted in black curves, with SnI_2_—in red curves.

The fragments of XRD patterns in the 2θ range 26–30° are depicted in Figure 7. These contain XRD peak 004 of perovskite and peak of FTO (SnO_2_) or the Cs_2_SnI_6_ phase. Peaks of FTO and perovskite are seen in Figure 7a in the case of the Bragg-Brentano mode. It can be seen that in the case of perovskite formed with the addition of SnI_2_, the XRD peak 004 is slightly shifted towards the higher diffraction angles. That should be the result of the replacement of some part of Pb ions by those of smaller Sn in the perovskite lattice. The lattice parameter *c* decreases due to this replacement and causes the peak shift [63].

Peaks 004 of perovskite and the Cs_2_SnI_6_ phase are visible on the pattern of perovskite formed with the addition of SnI_2_, in the instance of Pb-based perovskite, just the perovskite peak is visible. XRD peak 004 of the perovskite containing Sn, in contrast to the one of the Bragg-Brentano mode, is shifted towards lower diffraction angles, meaning the increased lattice parameter *c*. This can be caused by forming the Cs_2_SnI_6_ phase, which exhausts the smaller ions of Cs, Sn, and I compared to those of Pb and Br.

**Figure 8:**
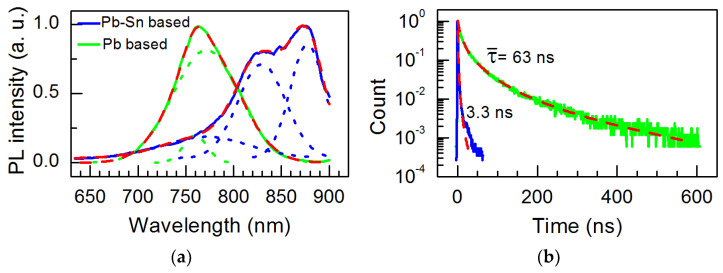
PL spectra (**a**) and transient decay (**b**) of the Pb-based (green) and Pb-Sn based (blue) perovskite layers. The red dashed lines are fitting results, and the dotted lines are Gaussian.

The PL spectra and transients measured at room temperature are shown in Figure 8. The peak of the Pb-based sample is fitted with two Gaussian functions. The difference is about 25 meV, close to the exciton binding energy determined from the absorption spectra [64]. The spectrum of the Pb-Sn based sample is fitted with three Gaussian peaks. One is fixed for the Sn-less sample, and two others are shifted to lower energies by 112 meV and 194 meV, respectively. The difference of 82 meV between them is too big to be related to the exciton binding energy. Thus, these two peaks could be related to the emission from differently sized crystallites or structures of different compositions [65,66].

We calculated numerically the average decay time as

(1) $$\bar{\tau }=\frac{\int_{0}^{\infty }tI_{\mathrm{PL}}(t)dt}{\int_{0}^{\infty }I_{\mathrm{PL}}(t)dt},$$

In this case, the transients were fitted using a generic decay function that included both the compressed hyperbola and stretched exponential [67].

(2) $$I_{\mathrm{PL}}(t)=Ae^{\frac{1-{\left(1+\alpha \frac{t}{\tau_{0}}\right)}^{\beta }}{\alpha \beta }},$$

where *α* and *β* are dimensionless parameters. The fitting results are also presented in Figure 8. One can see that the introduction of tin forms defects which increase the nonradiative recombination and decrease the average decay time from 63 ns to 3.3 ns. The capture and accumulation of photogenerated carriers to defects can change the voltage and decrease carrier density. Moreover, their subsequent release could maintain processes for a longer period of time. The photoluminescence lifetime acts as a precise indicator of the carrier dynamics within the perovskite material. An extended photoluminescence lifetime generally suggests that photogenerated carriers encounter fewer nonradiative pathways, allowing them to stay in excited states for a more extended duration, which aids in their extraction in optoelectronic devices. On the other hand, a reduced PL lifetime, as seen in the Pb-Sn system, indicates that carriers tend to recombine more quickly, frequently before they can be efficiently utilized for charge transport or emission.

**Figure 9:**
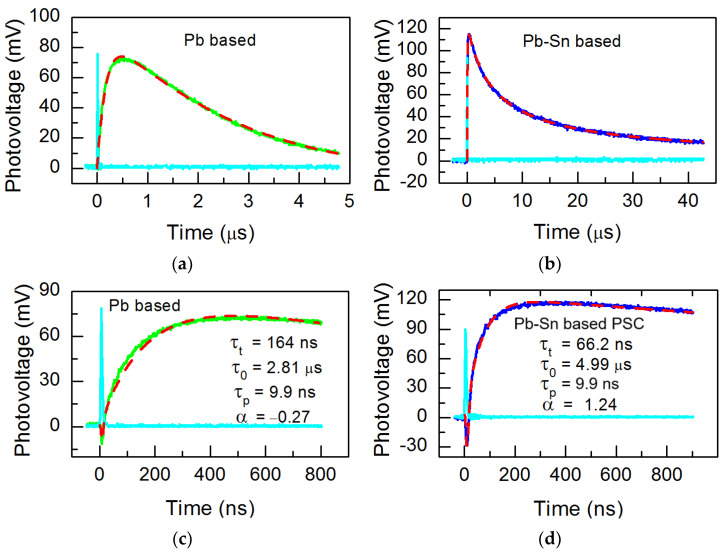
The transient photovoltage decay observed in solar cells constructed from Pb-based materials (**a**,**c**) and Pb-Sn based (**b**,**d**) perovskite films. Excitation laser power density is 0.6 mW/cm^2^ for Pb-based PSC and 0.8 mW/cm^2^ for Pb-Sn based PSC; the laser pulse is shown in cyan.

Figure 9 shows transient photovoltage decay of the solar cells fabricated on Pb based on mixed Pb-Sn perovskite films. The results of the photovoltage transients of PSCs show that the photovoltage decay times are longer than those of PL.

As Figure 9c,d show, the photovoltage consists of at least two components:

(3) $$U=U_{f}+U_{ph}.$$

The first, *U_f_*, is the fast photovoltage component with negative polarity and follows the laser pulse (cyan colored peak in all parts of Figure 9). This component arises as a result of the laser radiation-induced heating of free carriers. The shape of *U_f_* closely resembles that of the laser pulse and can be expressed as

(4) $$U_{f}=k_{f}I_{p}{\left(\frac{t}{\tau_{p}}\right)}^{4}\exp\left[-4\left(\frac{t}{\tau_{p}}-1\right)\right],$$

In this context, *I_p_* denotes the peak intensity of the laser, while *τ_p_* represents the rise time of the laser. The coefficient *k_f_* is derived from experimental measurements.

The second photovoltage component *U_ph_*, is slow. The typical photovoltage is a consequence of the generation of electron-hole pairs. The form of it can be articulated as [68]

(5) Uph=U0e−tτt−e−tτrec1τrec−1τt,

In this context, *U*_0_ represents the initial photovoltage, while *τ_t_* denotes the time constant associated with electron transport within the perovskite layer, and *τ_rec_* refers to the time required for carrier recombination.

**Figure 10:**
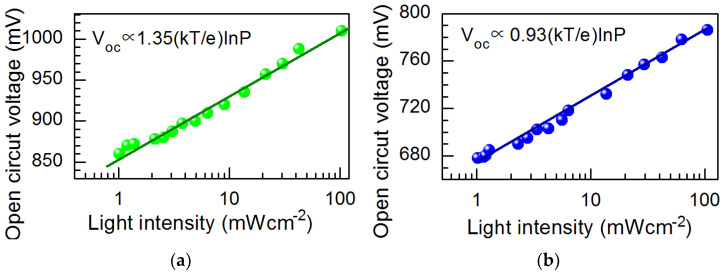
Dependence of open circuit voltage V_oc_ on white light intensity in solar cells fabricated on Pb-based (**a**) and Pb-Sn based (**b**) perovskite films.

The gradient of the open circuit voltage across the PSC in relation to the logarithm of light intensity elucidates the bimolecular recombination mechanism (refer to Figure 10). As this slope in our samples is close to one, we modified Equation (5) using a hyperbolic decay [69] as

(6) Uph=U0e−tτt−1+αtτ0−1α1τ0−1τt

where *τ*_0_ is the time constant at *t* = 0, *α* is a dimensionless parameter. Equation (6) turns to a compressed hyperbola if *α* < 1, to a stretched hyperbola if *α* > 1, and if *α* < 0, the integrated result becomes zero at a finite value of *t*. The results of the fitting were computed in accordance with Equations (4) and (6), and are illustrated in Figure 9 as dashed lines. The movement of excited charge carriers within perovskite films is predominantly influenced by their diffusion, given the relatively weak electric field present in this context. In this instance, D is the bipolar diffusion coefficient and L is the film’s thickness; therefore, *τ_t_* is roughly equal to L^2^/D. For Pb-based and mixed Pb-Sn perovskite films, respectively, the anticipated D values are 0.04 cm^2^/s and 0.15 cm^2^/s. These values of D are typical for spin-coated perovskite films [7,10,56,69,70].

Figure 10 shows the dependence of the open circuit voltage (V_oc_) on the white light intensity P. It is seen that V_oc_ of Pb-based PSCs is more significant than that of mixed Pb-Sn PSCs. This difference can be determined by a narrower forbidden energy band gap and dominant surface recombination of charge carriers in Pb-Sn perovskite films [27,62,63]. The latter effect is evidenced by the dependence of V_oc_ on illumination. The 0.93 *k*_B_*T*/*e* value of the slope of the open circuit voltage versus the logarithm of light intensity shows that the surface recombination of charge carriers is dominant in Pb-Sn perovskite films [71].

**Figure 11:**
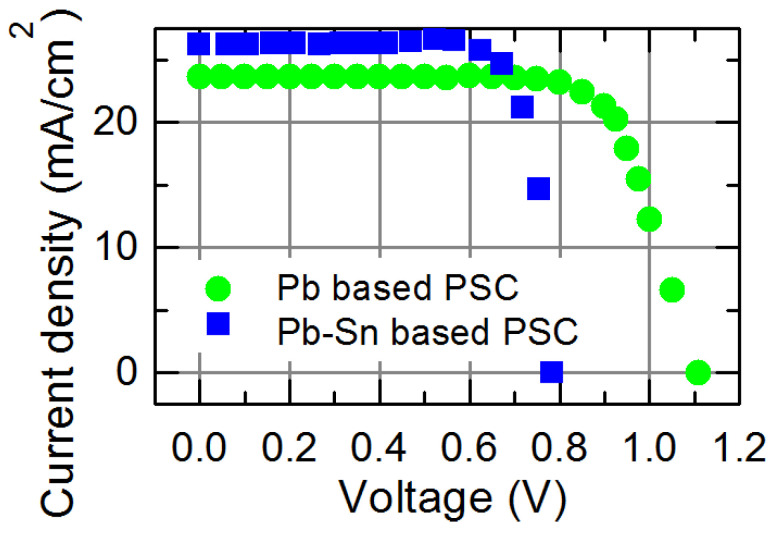
Current–voltage characteristics of the solar cells fabricated on Pb-based and Pb-Sn based perovskite films under 100 mW/cm^2^ of white light irradiance.

Moreover, affecting the PCE of solar cells is the lifespan of the produced charge carriers. Figure 11 shows the measured current–voltage characteristics of the best fabricated PSCs both with and without Sn. Table 1 lists the values of the corresponding photovoltaic quantities.

It is seen that the PCE of the Pb-Sn based perovskite solar cells is lower than that of Pb-based perovskite SC. The high value of open circuit voltage resulting from a larger forbidden energy gap compared to the Pb-Sn based perovskite sample mostly determines the high PCE of Pb-based PSC. In addition, the non-radiative recombination rate in the Pb-based perovskite is lower than in the mixed Pb-Sn perovskite films [63]. The value of PCE of Pb-based solar cell 19.1% is typical (16.5–19.2)% for triple cation perovskite SC [72]. The performance of mixed Pb-Sn triple cation perovskite SC varies over a wide range (14–18)% depending on the composition and measurement conditions [73,74]. The incorporation of tin into a triple cation perovskite film reduces the PCE of solar cells due to the increased effect of non-radiative defects [63] and surface recombination rate.

## 4. Conclusions

An experimental study of structural, photoluminescence, and optical properties of two type perovskite layers, Cs_x_(FA_0.83_MA_0.17_)_(1−x)_Pb(I_0.83_Br_0.17_)_3_ and Cs_x_(FA_0.83_MA_0.17_)_(1−x)_Pb_0.8_Sn_0_._2_(I_0.83_Br_0.17_)_3_, was carried out. The presence of the hot carrier effect and the influence of hot carriers on formation of photovoltage across perovskite solar cells was revealed by the measurements of transient photovoltage. It was found that the photovoltage consists of two components having opposite polarities. The fast component *U_f_* follows the laser pulse shape and is caused by charge carrier heating by light. The slow component is the usual photovoltage arising due to electron-hole pair generation. Since the polarity of *U_f_* is opposite that of the conventional photovoltage, the hot charge carriers can provide another reason restricting the PCE of PSCs. Since the value of the hot carrier thermoelectromotive force is directly proportional to the height of the potential barrier caused by band bending near the charge transport layers, the negative impact of hot carriers can be mitigated by reducing the band bending. In the case of a flat band, the hot carrier thermoelectromotive force vanishes. The introduction of tin into a perovskite reduces the PCE of solar cells due to the decreased forbidden energy gap and increased effect of non-radiative defects and surface recombination rate.

## Figures and Tables

**Table 1:** Pb and Pb-Sn incorporating PSC: photovoltaic parameters. The open circuit voltage is V_oc_; the short circuit current is J_sc_; the fill factor is FF; the PCE.

Perovskite Solar Cell	V_oc_, V	J_sc_, mA·cm^−2^	FF, %	PCE, %
Pb based	1.11	23.6	73	19.1
Pb-Sn based	0.785	26.2	70	14.4

## Data Availability

The original contributions presented in this study are included in the article. Further inquiries can be directed to the corresponding authors.
